# Cytotoxic and Apoptotic Activity of Majoranolide from* Mezilaurus crassiramea* on HL-60 Leukemia Cells

**DOI:** 10.1155/2019/3464237

**Published:** 2019-03-03

**Authors:** Lanna M. Heemann, Kamylla F. S. de Souza, Danilo Tófoli, Kelly J. Filippin, Walmir S. Garcez, Maria de Fatima C. Matos, Fernanda R. Garcez, Renata T. Perdomo

**Affiliations:** ^1^Laboratory of Molecular Biology and Cell Culture, School of Pharmaceutical Sciences, Food Technology, and Nutrition, Universidade Federal de Mato Grosso do Sul, Campo Grande, MS, Brazil; ^2^Institute of Chemistry, Universidade Federal de Mato Grosso do Sul, Campo Grande, MS, Brazil

## Abstract

Majoranolide, a butanolide isolated from the nonpolar fraction of an ethanol extract of* Mezilaurus crassiramea* (Lauraceae) fruits, is being reported for the first time in this genus and the third time in plants. Structurally identified from 1D and 2D NMR and HRESIMS data, majoranolide proved cytotoxic against cancer cells—MCF-7 and MDA-MB-231 (breast), HT-29 (colon), PC-3 (prostate), 786-0 (renal), and HL-60 (leukemia)—inhibiting growth in HL-60 cells (GI_50_ = 0.21 *μ*M) and exhibiting higher selectivity for this line than for nonneoplastic NIH/3T3 murine fibroblasts. Effects on the cell cycle, caspase-3 activation, and plasma membrane integrity were evaluated by flow cytometry. Expression of genes related to apoptotic pathways (*BAX*,* BCL2*,* BIRC5*, and* CASP8*) was investigated using RT-qPCR. At 50 *μ*M, majoranolide induced cell cycle arrest at G1 in 24 h increased the sub-G1 population in 48 h and increased caspase-3 activation in a time-dependent manner. The compound upregulated* BAX* and* CASP8* transcription (proapoptotic genes) and downregulated* BIRC5* (antiapoptotic). Loss of plasma membrane integrity in 30% of cells occurred at 48 h, but not at 24 h, characterizing gradual, programmed death. The results suggest that majoranolide cytotoxicity involves apoptosis induction in HL-60 cells, although other mechanisms may contribute to this cell death.

## 1. Introduction

*Mezilaurus crassiramea* (Meisn.) Taub. ex Mez. (Lauraceae), a tree commonly found in Mato Grosso do Sul state, Midwest Brazil (vernacular names: “canela-branca”, “canela-de-goiás”, “cumbuquinha”, “itaúba-abacate”), occurs frequently in Cerrado landscapes on the Brazilian Plateau [1].

Previous investigation of the activity of the species' leaf extracts against brine shrimp (*Artemia salina*) led to the isolation of three butanolides, namely, rubrenolide and its corresponding 2′- and 2′,3′-acetyl derivatives, that proved cytotoxic to human cancer cells [2].

Several compounds obtained from Lauraceae bearing a *γ*-butyrolactone backbone have received considerable attention for their potential cytotoxicity [3–6]. Poor specificity of pharmacological treatments has been a major obstacle to reducing morbidity and mortality associated with cancer, as most available therapies can also damage normal cells. Relapse and drug resistance are other problems affecting success in cancer therapy. These obstacles have stimulated the search for potent, selective molecules that can serve as anticancer drugs [7, 8]. Activating programmed death (apoptosis) constitutes a promising target in the development of more effective drugs for cancer treatment [9]. As part of our group's efforts to identify novel constituents with potential anticancer activity in* M. crassiramea*, the present investigation was conducted using fruits of this species. This article reports the isolation of majoranolide, a butanolide, and the investigation of its cytotoxicity against a panel of human neoplastic cell lines. An evaluation of the effects of the compound on the cell cycle of HL-60 cells and the apoptosis process demonstrated* in vitro* the anticancer potential of majoranolide.

## 2. Materials and Methods

### 2.1. General Experimental Procedures

Optical rotation was determined on a Perkin Elmer 341 polarimeter. HRESIMS data were acquired with electrospray ionization in positive ion mode on an UltrOTOF-Q instrument (Bruker Daltonics). NMR spectroscopic data were recorded at room temperature in CDCl_3_ (Cambridge Isotope Laboratories) on a Bruker DPX-300 spectrometer operating at 300.13 MHz (^1^H)/75.47 MHz (^13^C). Column chromatography procedures were performed on silica gel 60 (70-230 or 230-400 mesh; Merck) and Sephadex LH-20 (Amersham Pharmacia Biotech).

### 2.2. Plant Material

Fruits of* M. crassiramea* were collected from Campo Grande, Mato Grosso do Sul, Brazil, in August 2014. The plant material was identified by Professor Flavio Macedo Alves and Professor Arnildo Pott (Institute of Biosciences, Universidade Federal de Mato Grosso do Sul). A voucher specimen (no. 33014) has been deposited at the CGMS Herbarium of the Universidade Federal de Mato Grosso do Sul.

### 2.3. Extraction and Isolation

Unripe fruits (271 g) were cut and extracted with 95% EtOH at room temperature. The residue obtained from the bioactive EtOH extract was subsequently partitioned between MeOH-H_2_O (9:1) and hexane, MeOH-H_2_O (1:1) and CH_2_Cl_2_, and MeOH-H_2_O (1:1) and EtOAc. Part of the bioactive hexane phase (3.0 g, from a total of 4.3 g) was then chromatographed on a silica gel 70-230 mesh column (3 × 11 cm), using step gradient elution with hexane, hexane-EtOAc (25 → 75%), and EtOAc to give six fractions (A→F). An aliquot of fraction C (1.0 g, from a total of 1.4 g) was subjected to column chromatography on silica gel 230-400 mesh (2.5 × 22.5 cm), eluted with a gradient of hexane-EtOAc (5→50%), and EtOAc, followed by gel permeation column chromatography over Sephadex LH-20 (1.5 × 13 cm) eluted with CH_2_Cl_2_-MeOH (7:3) to yield majoranolide (62.0 mg).

### 2.4. Majoranolide

White amorphous powder; [*α*]_*D*_^23^ = –35.0° (*c* 0.1, acetone). ^1^H NMR (300 MHz, CDCl_3_): *δ* 0.82 (3H,* t*,* J* = 6.8 Hz, H-19), 1.20 20H,* brs*, H-9 to H-18), 1.36-1.48 (2H, m, H-8), 2.12 (2H,* q*,* J* = 7.6, H-7), 2.64 (1H,* brd*,* J* = 17.0 Hz, H-3a), 2.89 (1H,* dd*,* J* = 17.0, 8.4, H-3b), 3.58 (1H,* dd*,* J* = 12.4, 5.0, H-5a), 3.82 (1H,* dd*,* J* = 12.4, 3.0, H-5b), 4.55-4.65 (1H,* m*, H-4), 6.67 (1H,* tt*,* J* = 7.6, 3.0, H-6). ^13^C NMR (75 MHz, CDCl_3_): *δ* 14.1 (C-19), 22.6 (C-18), 26.7 (C-3), 29.3-29.6 (C-8 to C-16), 30.2 (C-17), 31.8 (C-7), 64.2 (C-5), 77.7 (C-4), 125.8 (C-2), 141.6 (C-6), 171.3 (C-1). HRESIMS (positive):* m/z *311.2594 [M+H]^+^ (calcd. for C_19_H_35_O_3_ 311.2586),* m/z *333.2402 [M+Na]^+^ (calcd. for C_19_H_34_O_3_Na 333.2406).

### 2.5. Sample Preparation

The test samples were dissolved in dimethylsulfoxide (DMSO) at 0.1 g/mL and stored at –20°C until experiment time, when they were diluted in culture medium containing antibiotics, but devoid of fetal bovine serum. The highest DMSO concentration employed, of 0.25%, does not affect cell viability.

### 2.6. Cell Culture

The ethanol extract, hexane phase, and majoranolide were evaluated for toxicity against six human neoplastic cell lines, namely, MCF-7 (breast, ATCC HTB-22), HT-29 (colon, ATCC HTB-38), PC-3 (prostate, ATCC CRL-1435), 786-0 (renal, ATCC CRL-1932), MDA-MB-231 (triple-negative breast, ATCC HTB-26), all of which were donated by Professor João Ernesto de Carvalho, of the School of Pharmaceutical Sciences, Universidade Estadual de Campinas, and HL-60 (promyelocytic leukemia, ATCC CCL-240), donated by Professor Fabíola Attié de Castro, of the Ribeirão Preto School of Pharmaceutical Sciences, Universidade de São Paulo. NIH/3T3 (murine fibroblast, ATCC CRL-1658) nonneoplastic cells were obtained from the Rio de Janeiro Cell Bank. Originally cryopreserved in liquid nitrogen, the cells were thawed for the tests and cultured in Roswell Park Memorial Institute (RPMI) 1640 medium or Dulbecco's Modified Eagle's Medium (DMEM) (the latter for NIH/3T3 cells) supplemented with 10% fetal bovine serum (Invitrogen) and amended with antibiotics to final concentrations of 100 IU/mL for penicillin and 100 *μ*g/mL for streptomycin (P4333 Sigma). The cells thus prepared were kept in an incubator at 37°C in a humidified atmosphere with 5% CO_2_ until test time.

### 2.7. Cytotoxicity Assay

For adherent cells, cytotoxicity was evaluated using the sulforhodamine B (SRB) dye [10]. For nonadherent cells (HL-60), the 3-(4,5-dimethylthiazol-2-yl)-2,5-diphenyltetrazolium bromide (MTT) dye was employed [11]. T_0_ (time zero) and T (test) plates were prepared using triplicate suspensions at densities of 3.5 × 10^3^ nonneoplastic cells, 7.5 × 10^3^ adherent neoplastic cells, or 2.5 × 10^4^ nonadherent neoplastic cells per well. T_0_ plates were incubated for 24 h and read on the day when the samples were added to the test plates in triplicate at four concentrations (0.25, 2.5, 25, and 250 *μ*g/mL). When the concentration that inhibits growth by 50% was lower than the smaller dose tested, the assay was repeated at concentrations 10 times lower, both for majoranolide and the positive control. The plates were then incubated for 48 h. Doxorubicin hydrochloride (2 mg/mL, Libbs Farmacêutica) was used as a positive control at a concentration 10 times lower than those of test samples. Forty-eight hours after addition of the test samples, the cells were fixed with 20% trichloroacetic acid and stained for 30 min with SRB (0.4%, w:v) dissolved in 1% acetic acid. The plates were subsequently washed four times with 1% acetic acid to remove excess dye and then dried. Trizma Base buffer (Sigma) at 10 mM, pH 10.5, was then added to solubilize the dyes bound to proteins in the fixed cells. For nonadherent cells (HL-60), addition of samples was followed, 48 h later, by addition of MTT to achieve a final concentration of 0.5 mg/mL in the test plates. These plates were then incubated for 4 h and received 100 *μ*L of DMSO. Absorbance was read on a SpectraMax 190 microplate spectrophotometer (Molecular Devices) at 540 nm for adherent cells and 570 nm for HL-60 cells. Absorbance readings, obtained in triplicate for each concentration, were used to calculate the means and standard deviations of growth percentages for each cell line, using SoftMax Pro 6.3 software, as per National Cancer Institute guidelines [12, 13]. From the growth percentages, GI_50_ values (corresponding to 50% growth inhibition) were calculated by nonlinear regression analysis using Origin 6.0 software. Selectivity indices were obtained as ratios between GI_50_ values found for nonneoplastic cells and GI_50_ values for neoplastic cells. Selectivity indices higher than 2.0 were considered significant [14].

### 2.8. Cell Cycle Analysis

HL-60 cells (5 × 10^5^ cells/well) were seeded in 12-well plates and incubated at 37°C in a humidified atmosphere with 5% CO_2_ for 24 h and subsequently exposed to one of four concentrations of majoranolide (0.5, 5, 10, or 50 *μ*M) for 24 or 48 h. The cells were then collected, centrifuged at 300* g* for 5 min, washed with PBS, and resuspended in membrane lysis buffer (0.1% Triton X-100, 0.1 mM EDTA, and 50 *μ*g/ml of RNAse in PBS), to which staining solution 7-AAD (BioLegend) was then added. After 30 min incubation, the content was measured on a BD Accuri C6 Plus flow cytometer and data were processed using FlowJo software.

### 2.9. Active Caspase-3 Quantification Assay

HL-60 cells (5 × 10^5^ cells/well) were seeded on 12-well plates and incubated at 37°C in a humidified atmosphere with 5% CO_2_ for 24 h and subsequently exposed to one of four concentrations of majoranolide (0.5, 5, 10, or 50 *μ*M) for 24 or 48 h. The cells were then collected, centrifuged at 300* g* for 5 min, washed with PBS, incubated in BD Cytofix/Cytoperm solution and kept on ice for 20 min. Two new washes with BD Perm/Wash buffer were performed and the resulting cell pellet was resuspended in 40 *μ*L of this buffer, to which PE Rabbit Anti-Active Caspase-3 antibodies were then added. This was followed by incubation for 30 min and a further centrifugation to remove excess reagent. Finally, the cells were resuspended in BD Perm/Wash buffer and the content was measured on a BD Accuri C6 Plus flow cytometer. Data thus obtained were processed using FlowJo software.

### 2.10. Quantification of mRNA Expression by RT-qPCR

Changes in* BAX*,* BCL2*,* BIRC5*, and* CASP8* mRNA expression by HL-60 cells were evaluated using RT-qPCR after treatment with 50 *μ*M majoranolide for 24 and 48 h. In a 6-well plate, 5 × 10^5^ cells were plated per well. After treatment, total RNA was extracted using a ReliaPrep RNA Cell Miniprep System kit (Promega), following manufacturer's instructions. RNA integrity was evaluated in 1% agarose gel. cDNA synthesis was performed using 1 *μ*g of total RNA and Oligo (dT)_15_ primer, employing a GoScript Reverse Transcription System kit (Promega), according to manufacturer's instructions. After synthesis, a 50 ng amount of cDNA was used for qPCR, together with SsoAdvanced Universal SYBR Green fluorophore (Bio-Rad). The reactions were carried out on a CFX96 Touch Real-Time thermal cycler (Bio-Rad) under the following thermocycling conditions: initial denaturation at 95°C for 30 s, 40 denaturation cycles at 95°C for 15 s, annealing at 62°C for 30 s, and extension at 72°C for 45 s. NTC and NRT controls confirmed the absence of contamination. Melt curve analysis confirmed the absence of nonspecific amplifications. Serial dilutions were performed to obtain the standard curve and efficiency values of primers for the genes investigated. The following primer sequences were employed:* BAX *forward: CGAGTGGCAGCTGACATGT, reverse: CAGCCCATGATGGTTCTG;* BCL2 *forward: TGGTGGAGGAGCTCTTCAG, reverse: TCAGGTACTCAGTCATCCAC;* CASP8 *forward: TGACCACGACCTTTGAAGAG, reverse: GAGAGGATACAGCAGATGAA;* BIRC5 *forward: CTAAGTTGGAGTGGAGTCTG, reverse: GGCTTGCTGGTCTCTTCTG;* β-ACTIN *forward: ACCCACACTGTGCCCATCTA, reverse: GGCAATGAGCGGTTCCG. Relative mRNA expression normalized to the *β*-actin gene was quantified using CFX Manager 3.1 software (Bio-Rad).

### 2.11. Cell Viability Evaluated with 7-AAD

HL-60 cells (5 × 10^5^ per well) were seeded on 12-well plates and incubated at 37°C in a humidified atmosphere with 5% CO_2_ for 24 h and subsequently exposed to one of four concentrations of majoranolide (0.5, 5, 10, or 50 *μ*M) for 24 or 48 h. The cells were then collected, centrifuged at 300* g* for 5 min, resuspended in PBS, and incubated in 7-AAD (BioLegend) for 15 min. The content was measured on a BD Accuri C6 Plus flow cytometer and data were processed using FlowJo software.

### 2.12. Statistical Analysis

Data were expressed as means ± SEM. One-way ANOVA was followed by Dunnett's posttest, to evaluate differences between treated and untreated cell groups, and Tukey's posttest, to determine differences between treated and untreated cells, as well as between treatment times. Statistical analysis was performed using GraphPad Prism 5.0 software. Differences were considered statistically significant when* p* < 0.05,* p* < 0.01, or* p* < 0.001.

## 3. Results and Discussion

The crude ethanol extract of* M. crassiramea* fruits was evaluated on six human neoplastic cell lines, namely, MCF-7 (breast), HT-29 (colon), PC-3 (prostate), 786-0 (renal), MDA-MB-231 (triple-negative breast), and HL-60 (promyelocytic leukemia), and a nonneoplastic murine line (NIH/3T3, fibroblast), revealing strong activity of the extract (GI_50_ = 0.25 *μ*g/mL) against HL-60 cells.

The extract was partitioned with hexane to yield a nonpolar phase. The molecular formula of the compound isolated after fractionation of the latter was established as C_19_H_34_O_3_, on the basis of its HRESIMS in the positive ion mode (*m/z* 311.2594 [M+H]^+^ and* m/z* 333.2402 [M+Na]^+^), requiring three degrees of unsaturation (see Figure S1 in the Supplementary Material).

**Figure 1:**
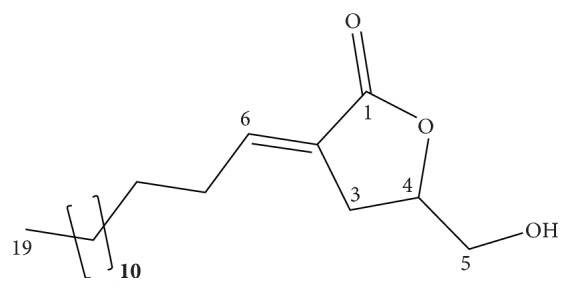
Chemical structure of majoranolide (molecular mass: 310.25 g/mol), a polyketide lactone.

The ^1^H NMR spectrum showed resonances attributable to a long linear alkyl chain (a broad singlet at *δ* 1.20 and a triplet at *δ* 0.82). A triple triplet suggestive of an olefinic hydrogen at *δ* 6.67 (*J* = 7.3 and 3.0 Hz), in addition to a pair of double doublets at *δ* 3.58 (*J* = 12.4 and 5.0 Hz) and 3.82 (*J* = 12.4 and 3.0 Hz), was also observed (see Figure S2 in the Supplementary Material). With the aid of information from the DEPT-135 spectrum, signals at *δ* 141.6, 125.8, and 171.3 in the ^13^C NMR spectrum were assigned to carbons of a trisubstituted double bond conjugated to a carbonyl lactone moiety (see Figures S3 and S4 in the Supplementary Material). Likewise, resonances at *δ*_C_ 77.7 (CH) and 64.2 (CH_2_) were ascribed to the carbon bearing the lactonic oxygen and to the carbon present in a hydroxymethylene residue, respectively. The foregoing data were thus consistent with the presence a butyrolactone-type skeleton bearing a conjugated exocyclic double bond and a hydroxymethylene group. Detailed analysis of the remainder of ^1^H and ^13^C resonances, as well as of connectivities discernible in COSY, HSQC, and HMBC spectra, led to identification of this butanolide as majoranolide (see Figure 1), whose published spectral data [15] were in full agreement with those obtained in the present study (see Figures S5–S8 in the Supplementary Material). Majoranolide, first isolated from* Persea major*, had its structure erroneously interpreted as that of a *δ*-lactone bearing a hydroxyl group at C-5 [16]. In 1996, the structure was revised as that of a *γ*-lactone containing an *α*-alkylidene-*γ*-hydroxymethylene moiety with (*E*)-geometry at the double bond [17].

Polyketide-derived *γ*-lactones bearing long *α*-alkylidene side chains, isolated from Lauraceae such as species pertaining to genera Aiouea, Lindera, Litsea, and Persea, are known for their cytotoxic properties [3–5, 16]. Majoranolide has been reported elsewhere [16] as causing 50% inhibition of cell growth in MCF-7 and HT-29 neoplastic cells when tested at 16.24 and 10.02 *μ*M, respectively. Drawing on this information, majoranolide isolated in the present study was evaluated for cytotoxicity against the six neoplastic cell lines employed in the assays with the ethanol extract of fruits, including MCF-7 and HT-29 cells previously tested elsewhere [16]. Majoranolide proved cytotoxic against these six cell lines, with strong activity against HL-60 cells (GI_50_ = 0.21 *μ*M) (see Table 1).

Although low specificity and side effects have been a major issue among anticancer agents [7], majoranolide proved at least 1000 times more selective for HL-60 cells than for NIH/3T3 nonneoplastic cells. The strong cytotoxic activity of majoranolide, as well as a differential cytotoxicity for leukemic HL-60 cells versus nonneoplastic cells, sparked our interest in evaluating the effect of the compound on cell death. This effect was investigated for 24 and 48 h treatments at four concentrations (0.5, 5.0, 10, and 50 *μ*M).

**Figure 2:**
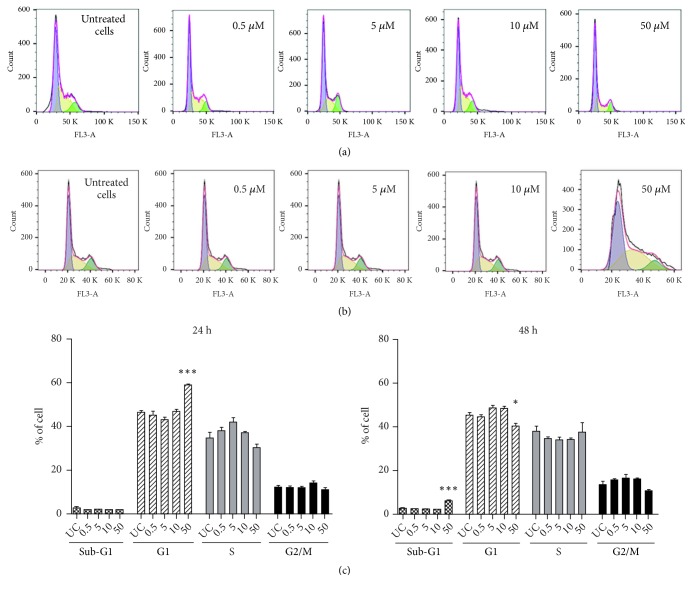
Effect of majoranolide on the cell cycle of HL-60 cells. After exposure to the compound (concentrations and times indicated), the cells were permeabilized, stained with 7-ADD, and counted on a BD Accuri C6 Plus cytometer. FlowJo software was employed for data analysis. Cell percentages at the G1, S, and G2/M phases were determined from histograms depicting DNA content* versus* number of untreated cells (UC) and treated cells after 24 h (a) and 48 h (b) treatments. (c) Results are shown as means ± SEM of three independent experiments. ^*∗*^*p* < 0.05; ^*∗∗∗*^*p* < 0.001 (one-way ANOVA followed by Dunnett's posttest).

Because cancer development involves deregulation of cell cycle control mechanisms, many chemotherapeutic agents act at specific phases of this cycle [18]. To investigate whether the cytotoxic effect of majoranolide was related to cell cycle arrest, HL-60 cells were treated with the compound at concentrations of 0.5, 5.0, 10, and 50 *μ*M for 24 and 48 h. To differentiate among cell populations in the G1, S, and G2/M phases, the cells were evaluated using flow cytometry after addition of DNA intercalator 7-AAD [19] (see Figure 2).

Employed at 50 *μ*M for 24 h, the compound induced cell cycle arrest at the G1 phase, with greater cell accumulation (68.86% ±1.02%) than controls (46.46% ±1.41%). At 48 h, an increase was observed in the sub-G1 population (6.31% ± 0.38%), characterizing fragmented DNA and apoptotic bodies [20]. Further evidence of apoptosis is provided by cell accumulation at the sub-G1 phase, indicating DNA damage. DNA damage activates a machinery of genes and proteins—such as p53, cyclins, and cyclin-dependent kinases (CDKs)—that acts to repair the damage. If the damage is irreversible, however, the cycle is arrested at the G1 phase, allowing apoptosis to take place [21, 22]. Two other butanolides isolated from Lauraceae (isophilippinolide A, from* Cinnamomum philippinense* (Merr.) Chang [23], and subamolide-E, from* C. subavenium *[24]) have also been shown to cause cell accumulation at the sub-G1 phase. The cytotoxic properties of butanolide derivatives from Lauraceae have been associated with the presence of an *α*-alkylidene-*γ*-lactone moiety that can act as a Michael-type acceptor for biological nucleophiles [25].

**Figure 3:**
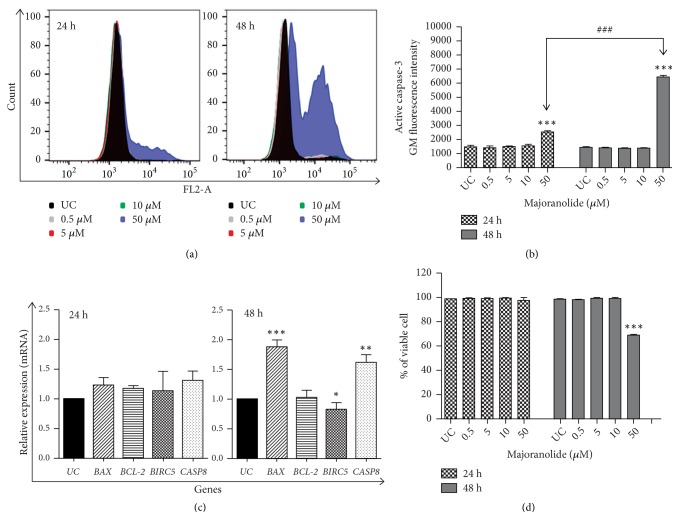
Effect of majoranolide on caspase-3 activation; expression of apoptosis-related genes and plasma membrane integrity in HL-60 cells. After cell exposure to the compound (concentrations and times indicated), the cells were stained with PE Rabbit Anti-Active Caspase-3 antibodies to quantify caspase activity or with 7-AAD to evaluate plasma membrane integrity, and then counted on a BD Accuri C6 Plus cytometer. FlowJo software was employed for data analysis. For analysis of gene expression, cDNA synthesis was performed from total RNA and data were collected using CFX Manager 3.1 software (Bio-Rad) and normalized to *β*-actin; untreated cells (UC) = 1. (*a*) Histograms depict fluorescence intensities of caspase-3 activity for treated and untreated cells after 24 h and 48 h treatments. (*b*) Geometric mean (GM) fluorescence intensity values. ^*∗∗∗*^*p* < 0.001 between treated and untreated cells; ^###^*p* < 0.001 between 24 and 48 h treatments (one-way ANOVA followed by Tukey's posttest). (*c*) mRNA relative expression by* BAX, BCL2, BIRC5*, and* CASP8 *shown as means ± SEM ^*∗*^*p* < 0.05; ^*∗∗*^*p* < 0.01; ^*∗∗∗*^*p* < 0.001 (one-way ANOVA followed by Dunnett's posttest). (*d*) Percentages of viable cells according to mean fluorescence intensity are expressed as means ± SEM. ^*∗∗∗*^*p* < 0.001 (one-way ANOVA followed by Dunnett's posttest).

Caspases play a key role in apoptosis. Lethal stimuli trigger a sequence of events leading to caspase-3 activation [26]. Acting as peptidases and cleaving specific targets for the release of other peptidases, caspase-3 causes organelles and cell molecules to break down [27], culminating in cell death by apoptosis. To explore the effect of majoranolide on caspase-3 activation, the enzyme's activity was quantified by flow cytometry. After exposure to the compound (four concentrations, two exposure times), the cells were permeabilized and anti-caspase-3 antibodies were added. As shown in Figures 3(a) and 3(b), majoranolide at 50 *μ*M induced time-dependent caspase-3 activation. Active caspase-3 increased 1.7-fold after 24 h and 4.4-fold after 48 h of treatment, compared with untreated cells.

To further investigate the apoptotic effects of majoranolide, changes in expression levels were also evaluated. To this end, RT-qPCR was performed to quantify transfected mRNA in cells treated with 50 *μ*M majoranolide, relative to untreated cells. Apoptotic genes of the intrinsic pathway (*BAX*,* BCL2*, and* BIRC5*) and extrinsic pathway* CASP8* were analyzed. At 24 h of treatment, no changes in mRNA expression were observed for any of the genes investigated (see Figure 3(c)). However, 48 h treatment caused upregulation in mRNA expression by apoptotic genes* BAX* and* CASP8*, in addition to downregulation of* BIRC5*.* BAX* encodes a protein of the intrinsic pathway of apoptosis that plays a role in pore generation in the mitochondrial membrane, allowing release of substances such as cytochrome* c*, apoptosis-inducing factor, and endonuclease G [28]. Our data show cycle arrest at the G1 phase at 24 hours, followed by a 1.8-fold increase in BAX expression in treated cells, relative to controls. After cycle arrest at G1, transcription factors such as p53 promote the transcription of genes involved in apoptosis, including* BAX *[29].

Expression of gene* CASP8 *increased by a factor of 1.6. In addition to directly activating caspase-3 (extrinsic pathway), caspase-8 activates caspase-3 via the intrinsic pathway after release of BID [30], the principal activator of BAX, a key protein in the intrinsic pathway [31, 32].* BIRC5 *and survivin, the protein this gene codifies, are directly related to cell survival [33]. By downregulating this gene, majoranolide promoted cell death. No changes were found in* BCL2* expression, a feature that corroborates the results obtained, since* BCL2* upregulation might have antiapoptotic effects.

Expression of gene* CASP8 *increased by a factor of 1.6. In addition to directly activating caspase-3 (extrinsic pathway), caspase-8 activates caspase-3 via the intrinsic pathway after release of BID [30], the principal activator of BAX, a key protein in the intrinsic pathway [31, 32].* BIRC5 *and survivin, the protein this gene codifies, are directly related to cell survival [33]. By downregulating this gene, majoranolide promoted cell death. No changes were found in* BCL2* expression, a feature that corroborates the results obtained, since* BCL2* upregulation might have antiapoptotic effects.

Because irreversible loss of plasma membrane integrity leads to cell death [34], the effect of majoranolide on membrane integrity was also evaluated using four concentrations and two treatment times. No changes were detected within 24 h, but 48 h of exposure to majoranolide at 50 *μ*M impaired plasma membrane integrity, as shown by a 30.8% increase in fluorescence on flow cytometry (see Figure 3(d)). Lower concentrations of the compound did not affect integrity.

Thus far, majoranolide had been obtained from only two species—*Lindera akoensis* and* Persea major* (both Lauraceae)—which makes this article the third report of the occurrence of this butanolide in plants and the first in the genus* Mezilaurus*.

## 4. Conclusion

Our results demonstrate that the cytotoxic effect of majoranolide induces apoptosis in HL-60 cells. This suggests that majoranolide can cause DNA damage, arresting cell division at the G1 phase, as demonstrated in the present study. Cell cycle arrest at this stage is related to the TP53 protein, a transcription factor of the* BAX* gene (intrinsic pathway), whose upregulation have been demonstrated. Upregulation of* CASP8 *expression was also evidenced, indicating involvement of the extrinsic apoptotic pathway. Activation of caspase-3, the main apoptosis-enforcing enzyme, followed by gradual loss of plasma membrane integrity, characterizes irreversible cell death. Furthermore, leukemic HL-60 cells are more sensitive than NIH/3T3 nonneoplastic cells to the cytotoxic effect of majoranolide. Further research on the cellular effects of majoranolide and other butanolides from Lauraceae is therefore needed to elucidate, for instance, possible involvement of multiple targets, so that the mechanisms of action of these compounds can be fully clarified.

## Figures and Tables

**Table 1:** * In vitro* cytotoxicity (GI_50_^*∗*^) of ethanol extract and majoranolide obtained from fruits of *Mezilaurus crassiramea*, as tested against neoplastic and nonneoplastic cells.

*Cells*	*Ethanol extract*	*Majoranolide*		*Doxorubicin*	
	*μ*g/mL	*μ*g/mL	*μ*M	*μ*g/mL	*μ*M
MCF-7	60.85±8.95	8.23±0.43	26.55±1.39	0.02±0.00	0.04±0.00
HT-29	>250	2.96±0.09	9.55±0.31	0.25±0.02	0.45±0.06
PC-3	>250	2.72±0.03	8.76±0.09	0.26±0.01	0.48±0.02
786-0	>250	22.73±0.35	73.26±1.15	0.02±0.00	0.04±0.00
MDA-MB-231	>250	24.36±0.18	78.51±0.58	0.19±0.01	0.36±0.02
HL-60	0.25±0.05	0.06±0.00	0.21±0.03	0.02±0.00	0.04±0.00
NIH/3T3	>250	70.61±10.97	227.59±35.37	0.29±0.02	0.54±0.04

^*∗*^GI_50_: concentration that inhibits cell growth by 50%. Doxorubicin was the positive control. Values represent means ± SD from three independent experiments.

## Data Availability

1D and 2D ^1^H and ^13^C NMR and HRESIMS data, as well as cell cytotoxicity, cycle analysis, caspase-3, mRNA expression quantification, and cell viability data used to support the findings of this study are included within the article or are within the supplementary information file. Additional information is available from the corresponding author upon request.

## Supplementary Materials

Supplementary Materials 1D- and 2D - ^1^H and ^13^C NMR spectra as well as HRESIMS data of majoranolide are available as Supporting Information.
