# Supplementary material for: Cytotoxic and Apoptotic Activity of Majoranolide from Mezilaurus crassiramea on HL-60 Leukemia Cells
Source: Evid Based Complement Alternat Med. 2019 Mar 3;2019:3464237. doi: 10.1155/2019/3464237 (PMC6421045; doi:10.1155/2019/3464237)
Supplement: Supplementary Materials — 1D- and 2D - 1H and 13C NMR spectra as well as HRESIMS data of majoranolide are available as Supporting Information. [file 3464237.f1.doc]

**SUPPLEMENTARY MATERIAL**

**Evidence-Based Complementary and Alternative Medicine**

**Cytotoxic and apoptotic activity of majoranolide from *Mezilaurus crassiramea* on HL-60 leukemia cells**

Lanna M. Heemann^1^, Kamylla F. S. de Souza^1^, Danilo Tófoli^2^, Kelly J. Filippin^1^, Walmir S. Garcez^2^, Maria de Fatima. C. Matos^1^, Fernanda R. Garcez^2^, Renata T. Perdomo^1^

^1^Laboratory of Molecular Biology and Cell Culture, School of Pharmaceutical Sciences, Food Technology, and Nutrition, Universidade Federal de Mato Grosso do Sul, Campo Grande, MS, Brazil.

^2^Institute of Chemistry, Universidade Federal de Mato Grosso do Sul, Campo Grande, MS, Brazil.

Correspondence should be addressed to Renata T. Perdomo;

e-mail: renataperdomo@gmail.com

**Figure S1.** HR-ESIMS (positive ion mode) of majoranolide.

**
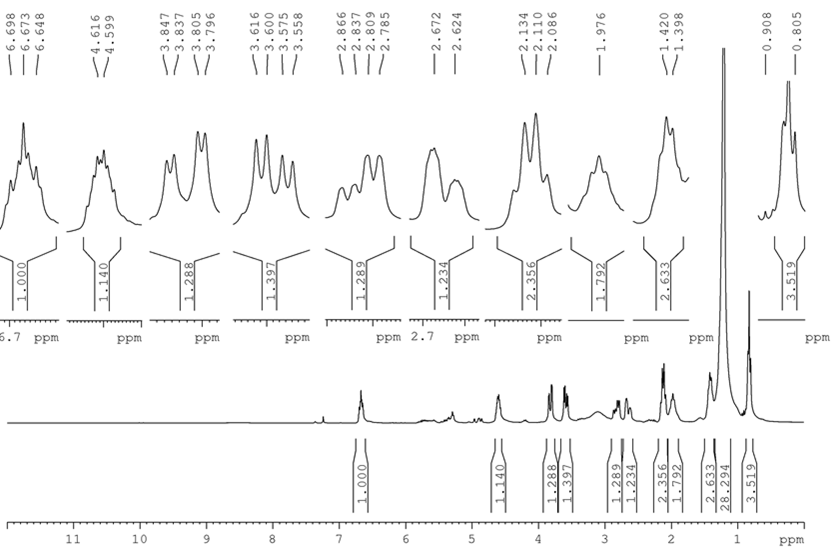
**

**Figure S2.** ^1^H (300 MHz, CDCl_3_) spectrum of majoranolide.


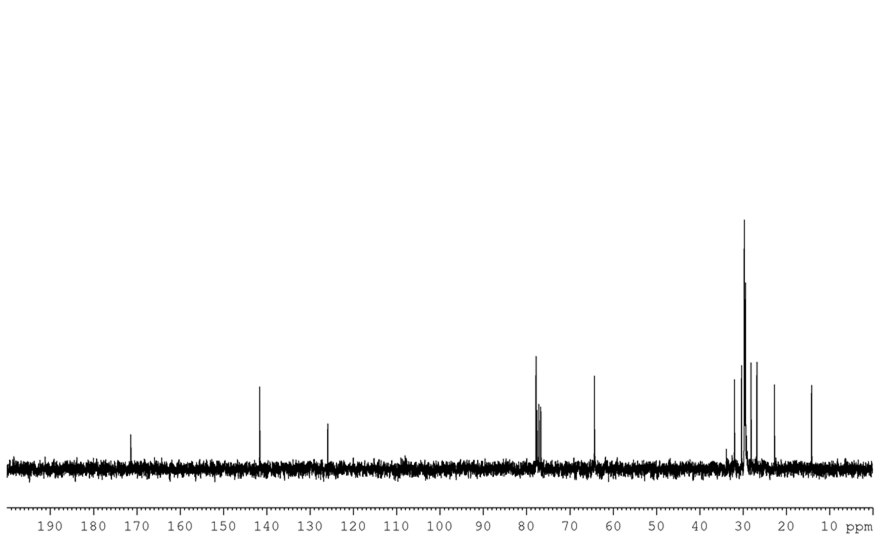


**Figure S3.** ^13^C (75 MHz, CDCl_3_) spectrum of majoranolide.


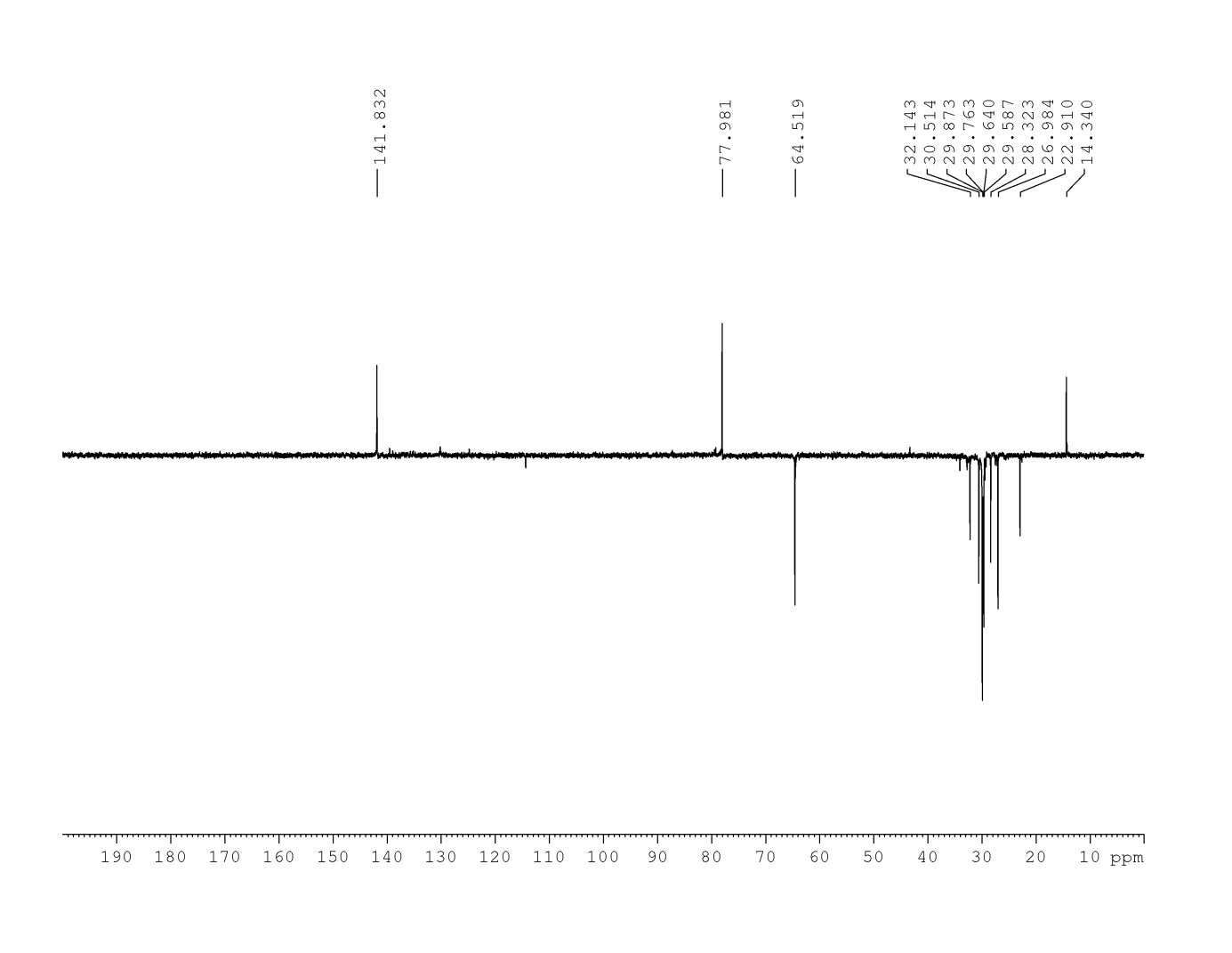


**Figure S4.** DEPT-135 experiment of majoranolide.


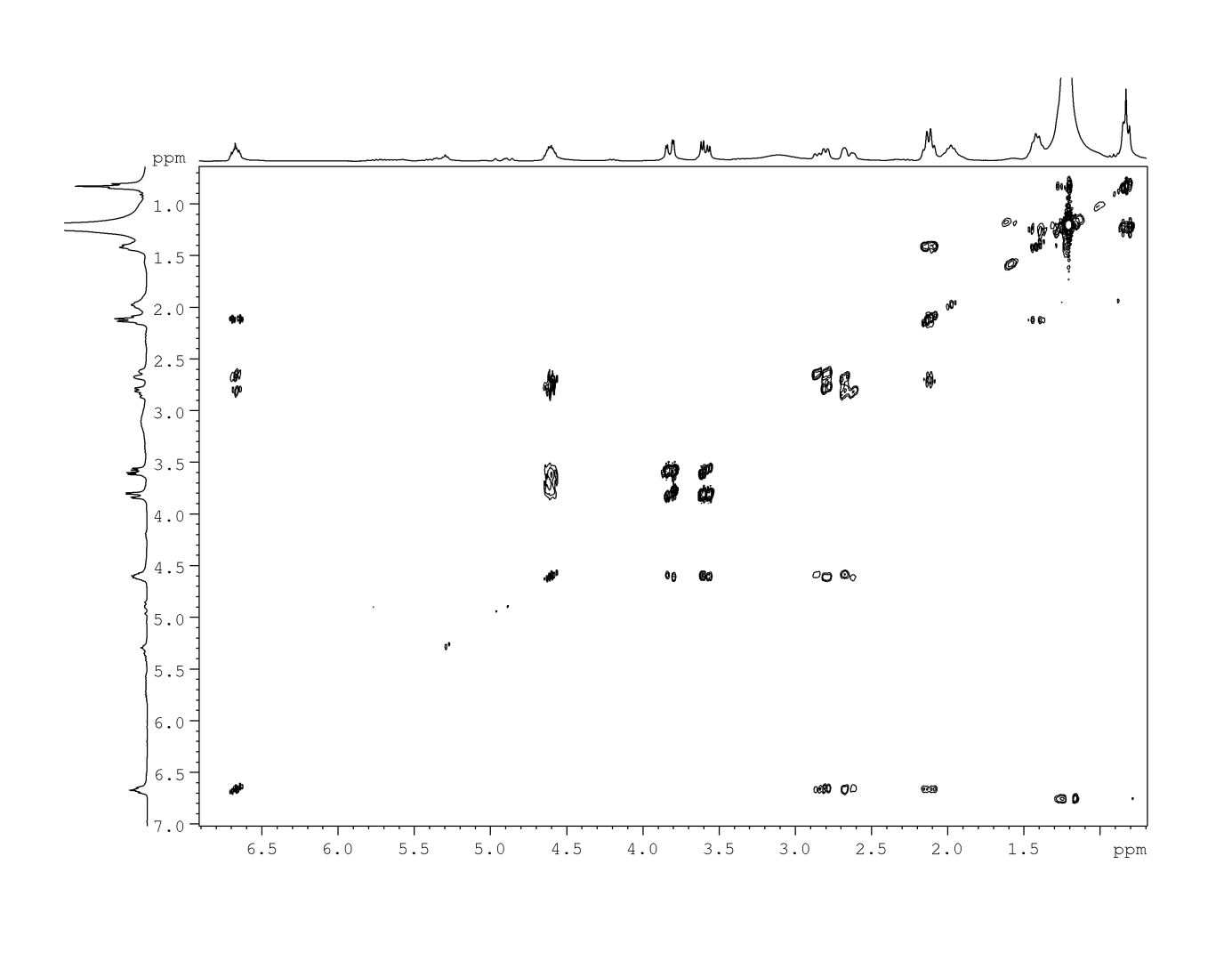


**Figure S5.** COSY experiment of majoranolide.


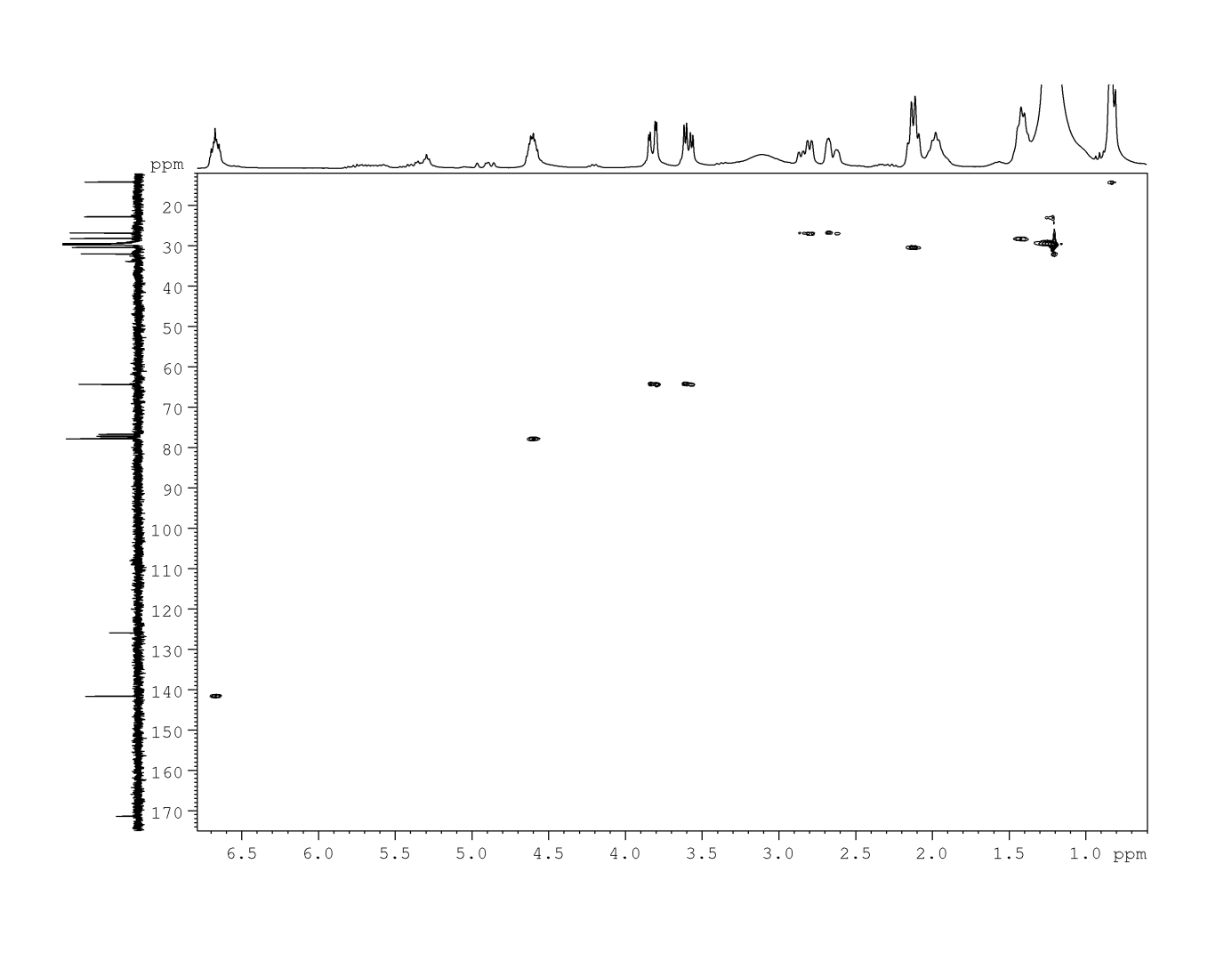


**Figure S6.** HSQC experiment of majoranolide.


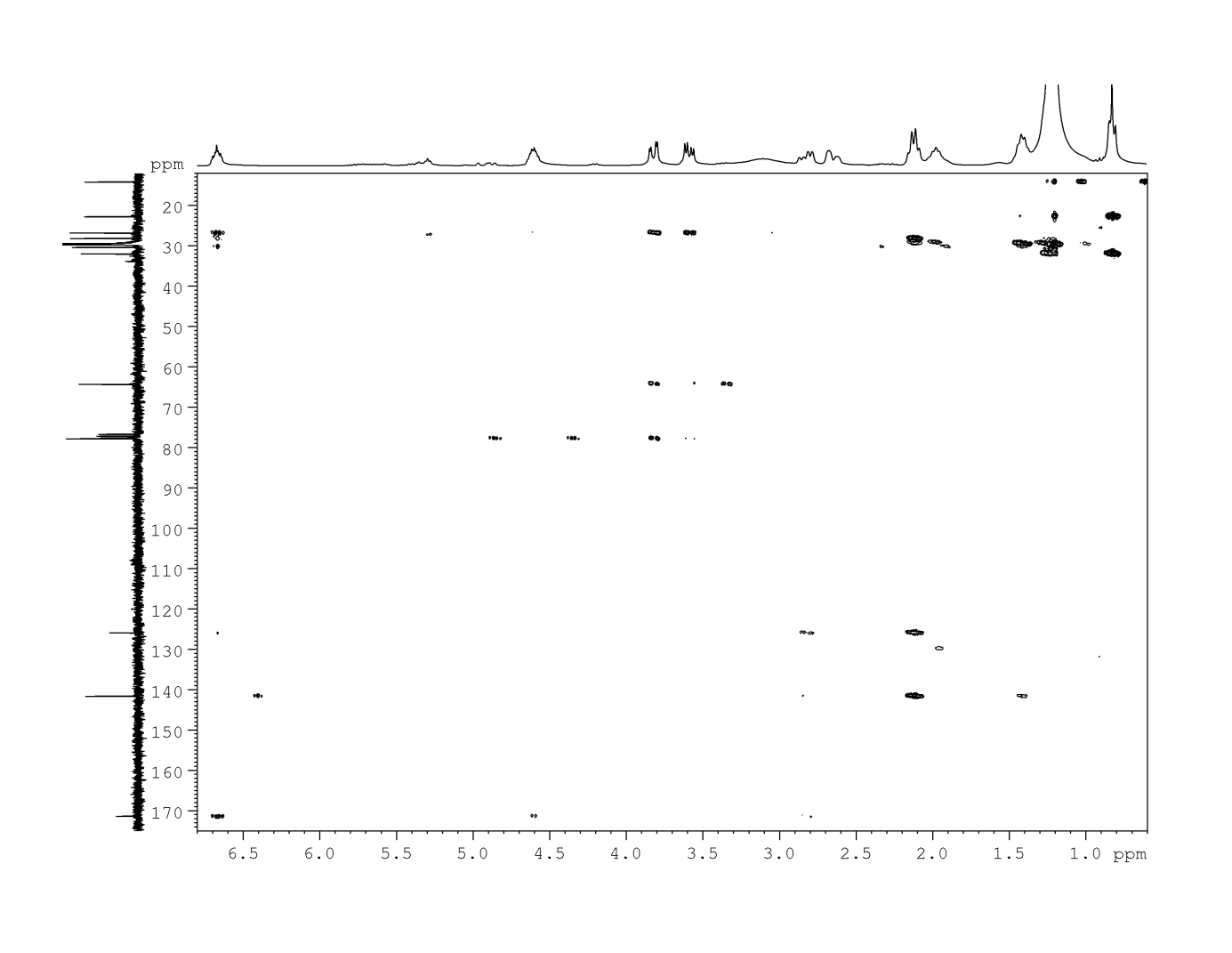


**Figure S7.** HMBC experiment of majoranolide.

**
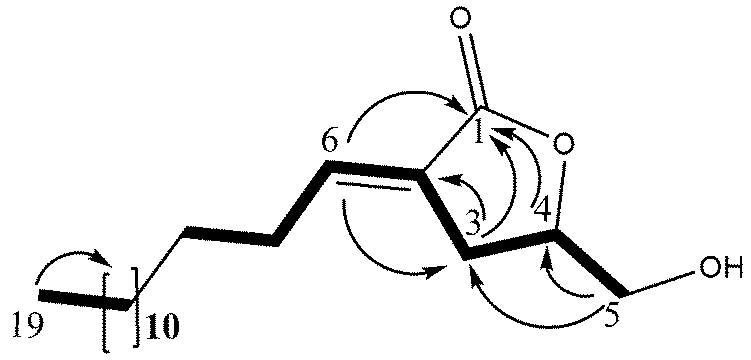
**

**Figure S8.** Key HMBC (→) and 1 H-1H COSY (**−**) correlations of majoranolide.
